# Rupture of the Uterus—Cæsarean Operation—Death in Seven Days

**Published:** 1862-12

**Authors:** L. R. Holmead

**Affiliations:** Chicago


### RUPTURE OF THE UTERUS—CÆSAREAN OPERATION—DEATH IN SEVEN DAYS.

Reported by L. R. HOLMEAD, M. D., Chicago.

Was called upon by Mr. B., Sunday, July 20, at 11 A. M., to see his wife in labor with her fifth child. On my arrival, found that she had been having pains since early in the evening previous. A midwife was in attendance. The pains said to have been slight until 3 A. M., when they became quite severe, and so continued until 9 A. M., when they ceased suddenly, the patient exclaiming, "What was that gave way?" and complaining of great distress in the abdomen.

All her former labors had been severe and prolonged. Upon examination, found the following condition: Pulse, 85 and full; nausea and retching, but no vomiting; no labor pains, but continued abdominal distress; head of child resting on floor of perineum. In fifteen or twenty minutes after my arrival, the pulse began to flag and vomiting commenced, but the latter was readily allayed by Opium. Fearing the nature of the trouble, I requested counsel, which, after some opposition on the part of the family, was acceded to. My friend, Dr. David Dodge, arrived at 12 M., and he agreeing with me, the family requested the further counsel of Dr. N. S. Davis, who came in at 2 P. M. He agreed with us in the propriety of instant removal of the child, although not decided as to nature of the trouble. The patient's pulse 120, and the head had receded slightly. Prof. Davis attempted to apply the forceps, but the child immediately receded. Upon examination it was found to have passed through the rupture, except the head and one arm. He then attempted to turn, but could not introduce his hand through the opening. Prof. Byford was then sent for, with request that he should come prepared to perform the Cæsarean operation. 4 P. M.—Patient growing more feeble; pulse 140.

4:30.—Prof. B. arrived. The Cæsarean operation decided upon and performed by him. Placenta and child both found in the peritoneal cavity; removed and wound closed in the usual manner with needles. 5:30.—Gave ½ gr. Sulph. Morph. and directed ¼ gr. to be repeated every hour during the night. 7:30 P. M.—Quiet, and seems to be getting under the influence of the narcotic. 8 o'clock.—Prof. B. called; pulse 130, weak. 10:30.—Sleeping; pulse same; skin moist.

21st, 2 P. M.—Continues in about same condition. Commenced giving Brandy ℥ ss every hour. 4 A. M.—About the same. 6 A. M.—Begins to be narcotized; pulse and skin in same condition. 8 A. M.—Prof. B. called; pulse rather more full; otherwise no change; brandy suspended. 12 M.—Abdominal muscles begin to relax; abdomen slightly tympanitic. 2 P. M.—Prof. B. again present; pulse more frequent; tympanitis increasing; feels weaker. Morphine continued. Tr. Veratrum Viride gtt iv.

6 P. M.—Apparently sinkiug; pulsc 150; very weak. Exhihited Morph. Sulph. and two drops Verat. Virid.

7 P. M.—Seems to be sinking rapidly. 8 P. M.—If any change, slight improvement. Prof. B. again present; ordered Turpentine gtt. iv., with Tinct. Opi gtt. x. in emulsion, every hour.

10 P. M.—Pulse getting more volume. 12.—Continued improvement. Comes out from under influence of opiates. Pulse 130, full. Complains of tenderness in the abdomen.

July 22nd.—Continued apparent improvement when examined at 2, 4 and 6 A. M. At 8 o'clock, Drs. Byford and Dodge came in; condition good; pulse 112. At 2 P. M., begins to give way again; nausea; pulse 130, and weaker. Discontinued Turpentine on account of the nausea; Morphine and Verat. Virid. continued. 8 P. M.—Had been rather more comfortable during the afternoon. At 11 P. M., tympanites began to diminish.

July 23d.—Has passed considerable gas with relief. The wound looks healthier this morning and traces of pus.

2 P. M.—Passed the morning comfortably; pulse not quite so good; tympaniets increasing. Ordered Quinine 3 grs. every four hours. Attempt to pass a rectum tube, but it will not go by the uterus. When removed, the orifice was found clogged with fæces. 6 P. M.—Considerable company has been permitted by the family. Patient is restless; tympanits increasing. Morph. gr. ss.

12 M.—Great excitement. Pulse 150, pretty full; abdomen enormously distended. Drs. Dodge and Byford present. Ordered injection Warm Water ℥ vj; Castor Oil ʒ ij; Turpertine ʒ j. M. Which moves off a large amount of gas, but no fæcal matter. This gave much relief, and the pulse lessened in frequency.

July 24th, 8 A. M.—Tympanites subsiding; has passed gas freely. Essence of beef ℥ ss, every two hours.

2 P. M.—Pulse 130; wound looking well. 8 P. M.—Has been sleeping quietly; tympanitis less.

July 25th.—Afterpains when the wound is dressed. 12 M. Spontaneous and free movement of the bowels; pulse somewhat excited. 2 P. M.—Pulse 130; no sleep. 8.—Symptoms of suppuration. Essence of beef ℥j, every two hours. No further movement of the bowels. After-pains becoming regular; discharged one clot; lochia free; no sleep. *Left parotid somawhat tumefied.*

July 26th, 8 A. M.—Not as well; had a bad night; the bowels had moved once; pulse 140. Swelling of the left parotid region increasing. Ordered: ℞. Comp. Tinct. Cinchonæ ʒj; Quiniæ Sulph. gr. j; Acid. Sulph. Aromat. gtt. v. M. To be given every two hours. Needles removed from the wound, which is looking badly. Adhesive plaster substituted.

2 P. M.—Worse. Diarrhœa. Ordered Tinct. Opi ʒj at once, and ʒss every two hours; Tinct. Cantharides gtt. xl ; but, on account of the difficulty of swallowing, the patient did not take it until four o'clock. 6 P. M.—Pulse fuller, 130; can swallow more readily. 10 P. M.—Pulse failing; parotid increasing in size; painted it with Tinct. Iodini; bowels still moving too freely, and wound looks badly.

July 27th, 8 A. M.—More feeble; diarrhœa continues. Continue treatment.

10 A. M.—No pulse at the wrist; cold to the elbows. Brandy *ad lib.* 11 o'clock.—Pulse returns; hands warm. 12 as at 11. 2½ P. M.—Sinking. At 6 P. M., in my absence, she was seized with convulsions. Drs. Byford and Dodge exhibit Chloroform, with relief. From this time she sunk rapidly. At 8, the spasms recurred; Chloroform produces vomiting, and death occurred at 9 o'clock P. M., being over seven days from the operation.

The single but pregnant comment upon this case is that, although it ultimately resulted fatally, nevertheless it contains within itself ample evidence that the formidable operation involved is warranted as a dernier resort in instances of this terrible accident.

